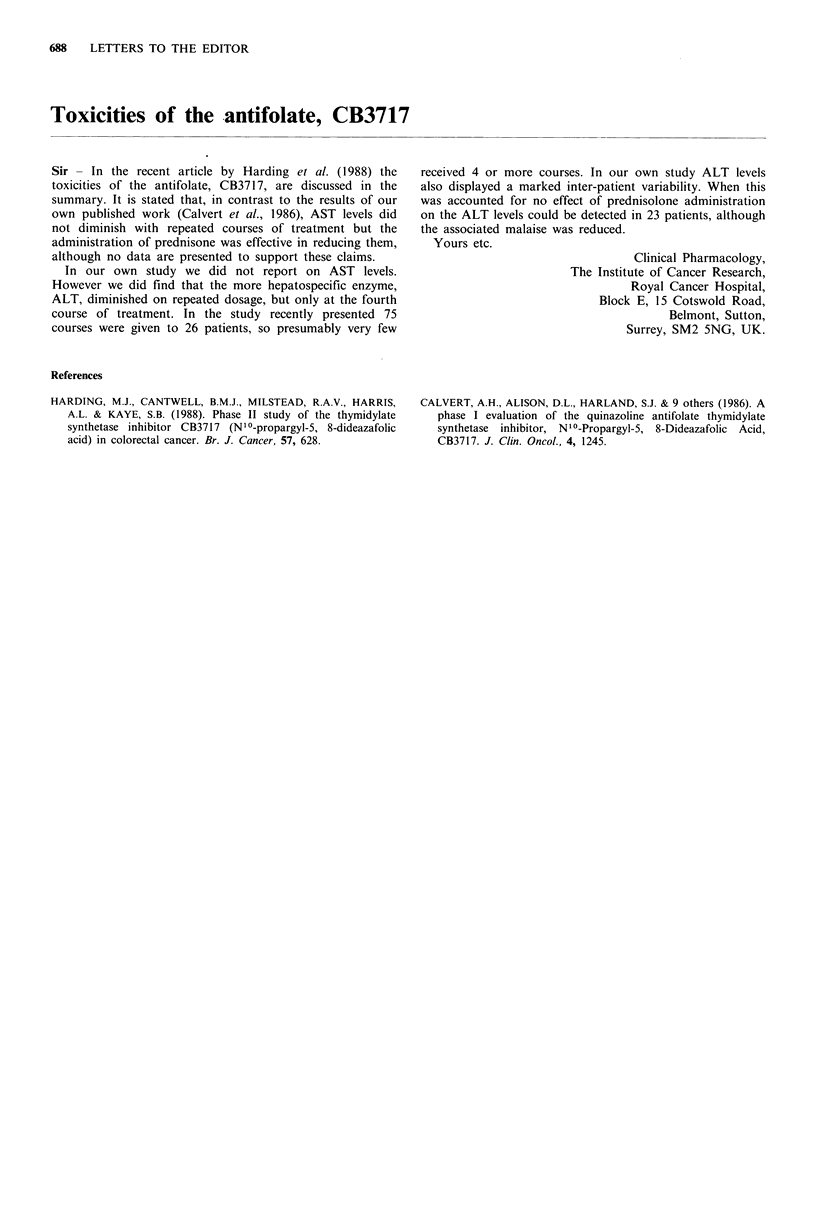# Toxicities of the antifoliate, CV3717

**Published:** 1988-11

**Authors:** 


### Toxicities of the antifolate, CB3717

**Sir** – In the recent article by Harding *et al.* (1988) the toxicities of the antifolate, CB3717, are discussed in the summary. It is stated that, in contrast to the results of our own published work (Calvert *et al.*, 1986), AST levels did not diminish with repeated courses of treatment but the administration of prednisone was effective in reducing them, although no data are presented to support these claims.

In our own study we did not report on AST levels. However we did find that the more hepatospecific enzyme, ALT, diminished on repeated dosage, but only at the fourth course of treatment. In the study recently presented 75 courses were given to 26 patients, so presumably very few received 4 or more courses. In our own study ALT levels also displayed a marked inter-patient variability. When this was accounted for no effect of prednisolone administration on the ALT levels could be detected in 23 patients, although the associated malaise was reduced.

Yours etc.

Clinical Pharmacology,
The Institute of Cancer Research,
Royal Cancer Hospital,
Block E, 15 Cotswold Road,
Belmont, Sutton,
Surrey, SM2 5NG, UK.

**References**

HARDING, M.J., CANTWELL, B.M.J., MILSTEAD, R.A.V., HARRIS, A.L. & KAYE, S.B. (1988). Phase II study of the thymidylate synthetase inhibitor CB3717 ($N^{10}$-propargyl-5, 8-dideazafolic acid) in colorectal cancer. *Br. J. Cancer,* **57,** 628.

CALVERT, A.H., ALISON, D.L., HARLAND, S.J. & 9 others (1986). A phase I evaluation of the quinazoline antifolate thymidylate synthetase inhibitor, $N^{10}$-Propargyl-5, 8-Dideazafolic Acid, CB3717. *J. Clin. Oncol.,* **4,** 1245.